# Supplementary material for: Development and Validation of the War Worry Scale (WWS) in a Sample of Italian Young Adults: An Instrument to Assess Worry About War in Non-War-Torn Environments
Source: Eur J Investig Health Psychol Educ. 2025 Feb 9;15(2):24. doi: 10.3390/ejihpe15020024 (PMC11853760; doi:10.3390/ejihpe15020024)
Supplement: Supplementary file 1 [file ejihpe-15-00024-s001.zip › Supplemental Material I.pdf]

Supplemental Material I

Narrative extracts used in the items generation of the Worry War Scale (WWS)

Giorgio Maria Regnoli & Barbara De Rosa

Content Analysis (Krippendorff, 1980) was carried out on a large amount of narrative material collected in December 2023 and January 2024 with a group of 200 young Italian adults (*M* = 21.10; *SD* = 2.09; 69.0% females; 30.5% males; 0. 5% non-binary) according to the following steps: 1) familiarization with the collected narrative material; 2) each researcher independently identified the thematic and semantic categories; 3) cross-checking of the identified thematic categories was carried out with the help of an independent judge.

Some narrative excerpts included in the emerging macro-theme "A Polytraumatic Era" but specifically related to the thematic subcategory "Worry about War" are presented below. The narrative excerpts were used to design items for the War Worry Scale (WWS). The items thus generated were supplemented by contributions from the literature on the impact of war, surveys conducted in the European context, and existing instruments on the construct of interest (deductive approach) [Boateng et al., 2018].

Narrative Extracts – Theme "Worry about War"

"Mi preoccupa il rischio di una guerra mondiale"
["I am worried about the risk of a world war."]

"[Sono preoccupat*] che si ritorni ai tempi disumani della guerra e che gli uomini inizino a vedere l'altro come nemico a cui sottrarre tutto e non una spalla per andare avanti".
["[I am worried] that we will return to the inhuman times of war and that people will begin to see the other as an enemy to be robbed of everything and not as a shoulder to stand on"]

"Una mia preoccupazione a livello collettivo riguarda sicuramente la guerra in Ucraina. In particolare, mi preoccupa che la maggior parte delle persone, giovani soprattutto, stanno scappando dal loro stesso Paese pur di non combattere e non supportare una guerra che non riguarda i loro valori e i loro ideali. Mi preoccupa che questa guerra faccia sì che tutti si concentrino solo sul punto di vista degli ucraini ma che non considerino come vittime anche i giovani russi che si trovano a scappare dalle loro terre e dalle loro famiglie e a non essere accettati da molti paesi"
["A collective worry of mine is definitely about the war in Ukraine. In particular, I am worried that most people, especially young people, are running away from their own country, in order not to fight and not to support a war that has nothing to do with their values and ideals. It worries me that this war makes everyone focus only on the Ukrainians' point of view, but that they do not also consider as victims the young Russians who are fleeing their country and their families and who are not accepted by many countries"]

“Sebbene non mi tocchi direttamente in quanto non ho difficoltà economiche, sono preoccupat* dell’aumento generale dei prezzi a causa della guerra Russo-Ucraina”

[“Although it does not affect me directly, as I have no economic difficulties, I am worried about the general increase in prices due to the Russo-Ukrainian war”]

“Dei problemi che non hanno un impatto diretto sulla mia vita, come la guerra in Ucraina, non riesco a preoccuparmi se non nel momento in cui focalizzo l’attenzione sul problema, attraverso articoli di giornale, foto o video che vedo su Internet. Quando mi rendo conto della situazione inizio a preoccuparmi”.
[“Of the problems that do not directly impact my life, such as the war in Ukraine, I cannot worry except at the moment when I focus my attention on the problem, through newspaper articles, photos or videos that I see on the Internet. When I become aware of the situation, I start to worry”].

“[Riferendosi alla guerra in Ucraina] sono preoccupato che possa accadere qualcosa del genere qui o che essa possa avere ripercussioni sul nostro paese e sulle persone che amo.

“[Referring to the war in Ukraine] I am worried that something like that could happen here or that it could affect our country and the people I love].

“Ho timore di una terza guerra mondiale [...]”.

[I am afraid of a third world war [...]]

“Sono preoccupata per i fatti che si sentono giornalmente al telegiornale. Sono preoccupata per la guerra, per la povertà nel mondo, credo che l'uomo sia capace di azioni deplorevoli [....]”.
["I am worried about the facts that we hear in the news every day. I am worried about war, about poverty in the world, I believe that people can do deplorable things [....]”]

“[...] Nessuno, o molto pochi si ricordano degli altri. Si parla di guerra perché per “colpa” della guerra mancano risorse o sono aumentati i costi di determinati beni, ma pochi si ricordano che dall’altra parte ci sono persone, esseri umani e soprattutto vittime come i bambini che non hanno nessuna colpa ma, nonostante ciò, vivono l’inferno ogni giorno”.

[“[...] No one, or very few, remember the others. People talk about war because there is a shortage of resources or an increase in the cost of certain goods due to the 'fault' of war, but very few remember that on the other side there are people, human beings, above all victims, such as children, who are not to blame but who nevertheless live in hell every day”]

“[...] In particolare mi preoccupa la guerra in Ucraina che dura da due anni. Si tratta di un evento che ha scosso l’intera umanità, ma soprattutto l’occidente che da tempo non pensava più tanto alla guerra, anche che si tratta in realtà di vero e proprio terrorismo. La mia preoccupazione è, dunque, sull'esito di questa guerra”.
[“[...] I am particular worried about the two-year war in Ukraine. It is an event that has shaken the whole of humanity, but especially the West, which for a long time has not thought so much about war, even about the fact that it is really terrorism. I am therefore worried about the outcome of this war”.]

“C’è la guerra, l’Iran, l’emergenza climatica, il Covid”.

[“There is the war, Iran, the climate emergency, Covid.”]

“[Mi preoccupa] il fatto che ad oggi ci si dichiari ancora guerra”.

[“[I am worried] that to this day we are still declaring war on each other.”]

“Mi preoccupa sapere se ci saranno altre guerre o anche altri fenomeni ambientali disastrosi. Mi preoccupa sapere se si riuscirà ad affrontare la crisi economica o ci sarà un suo peggioramento. Come si sopravviverà?”

[“I worry about whether there will be more wars or even other catastrophic environmental phenomena. I worry about whether the economic crisis will be overcome or worsened. How will people survive?”]

“Mi preoccupa la facilità con la quale si commettono atti atroci e che sono sempre più frequenti (stupri, guerre, omicidi)”.

[“I am worried about the ease with which heinous acts are being committed and are become more frequent (rape, war, murder)”]

“Le mie preoccupazioni principali vanno verso la nostra società e il mondo in cui viviamo. Le continue guerre, violenze e discriminazioni fanno pensare che non ci sia in realtà un bel futuro specialmente per noi giovani”.

["My main worries are about our society and the world we live in. The continuous wars, violence and discrimination make people think that there is actually no good future especially for us young people”]

“L'altra preoccupazione maggiore, in questo momento, è la situazione della guerra tra Russia/Ucraina/NATO che già finora ha influenzato le nostre vite, ma che potrebbe influenzare il nostro futuro in modi che ancora non sappiamo”

[“The other major worry at the moment is the situation of the Russia/Ukraine/NATO war, which has already affected our lives so far, but could affect our future in ways we don't yet know.”]

“Ho paura che questo mondo non sarà buono come un tempo e che i sovraccarichi che abbiamo per colpa delle guerre ci mettano in ginocchio”

[“I worry that this world will not be as good as it used to be and that the overload of wars will bring us to our knees”]

“[Mi preoccupa] il possibile utilizzo di armi nucleari per i prossimi conflitti in scala mondiale”

[“[I am worried about] the possible use of nuclear weapons in the coming global conflicts”]

The narrative material described is intended to support understanding of the process of developing the War Worry Scale (WWS) and cannot be used for other purposes as it is part of a larger ongoing research project (Scientific project manager: G.M. Regnoli; Approval Code: protocol number 1-2023; Approval Date: January 13, 2023; University of Naples Federico II).
