# Supplementary material for: Development and Validation of the War Worry Scale (WWS) in a Sample of Italian Young Adults: An Instrument to Assess Worry About War in Non-War-Torn Environments
Source: Eur J Investig Health Psychol Educ. 2025 Feb 9;15(2):24. doi: 10.3390/ejihpe15020024 (PMC11853760; doi:10.3390/ejihpe15020024)
Supplement: Supplementary file 1 [file ejihpe-15-00024-s001.zip › Supplemental Material II.pdf]

**Supplemental Material II**

**Items Excluded**

1. I’m worried about the expansion of wars in the world.
   (Mi preoccupa l’espansione delle guerre nel mondo)
3. I am worried about the effects of war on politics, the economy, and society.
   (Sono preoccupat* per gli effetti della guerra sulla politica, sull’economia, sulla società)
7. I'm worried that wars are making the world a dangerous place to live
   (Una mia preoccupazione è che le guerre stiano rendendo il mondo un posto pericoloso)
12. I'm worried to think of chemical and/or nuclear weapons getting used in the current conflicts.
    (Il pensiero che nei conflitti in corso si possa far uso di armi chimiche e/o nucleari mi preoccupa)
13. I worry that I cannot go to many parts of the world because of the wars.
    (Mi preoccupa non poter più andare in molte aree del mondo a causa delle guerre)
15. I worry that people will be forced to leave their countries because of the war.
    (Una mia preoccupazione è che le persone siano costrette a lasciare il loro paese a causa della guerra)
16. I am worried that information about the war is incomplete, unreliable, or biased.
    (Mi preoccupa che le informazioni sulla guerra siano incomplete, inattendibili o di parte)
